# Supplementary material for: Proposed Mechanism for the Antitrypanosomal Activity of Quercetin and Myricetin Isolated from Hypericum afrum Lam.: Phytochemistry, In Vitro Testing and Modeling Studies
Source: Molecules. 2021 Feb 14;26(4):1009. doi: 10.3390/molecules26041009 (PMC7918497; doi:10.3390/molecules26041009)
Supplement: Supplementary file 1 [file molecules-26-01009-s001.pdf]

Proposed Mechanism for the Antitrypanosomal Activity of Quercetin and Myricetin isolated from *Hypericum afrum* Lam.: Phytochemistry, in Vitro Testing and Modeling Studies

Farida Larit [1,2,*], Khaled M. Elokely [3,4], Manal A. Nael [3,4], Samira Benyahia [5], Francisco León [2,6], Stephen J. Cutler [2,6] and Mohammed M. Ghoneim [7,8,*]

1 Département de Chimie, Faculté des Sciences Exactes, Université des Frères Mentouri Constantine 1, Constantine 25000, Algeria
2 Department of BioMolecular Sciences, Division of Medicinal Chemistry, University of Mississippi, University, MS 38677, USA
3 Department of Pharmaceutical Chemistry, Faculty of Pharmacy, Tanta University, Tanta 31527, Egypt
4 Institute for Computational Molecular Science, Department of Chemistry, Temple University, Philadelphia, PA 19122, USA; kelokely@temple.edu (K.M.E.); mnael@pharm.tanta.edu.eg (M.A.N.);
5 Laboratoire de Synthèse Organique, Modélisation et Optimisation des Procèdes (LOMOP), Université Badji Mokhtar, 23000 Annaba, Algeria; samira.benyahia13@gmail.com
6 Department of Drug Discovery and Biomedical Sciences, College of Pharmacy, University of South Carolina, Columbia, SC 29208, USA; jleon@mailbox.sc.edu (F.L.); sjcutler@cop.sc.edu (S.J.C.)
7 Department of Pharmacy Practice, College of Pharmacy, AlMaarefa University, Ad Diriyah, Riyadh 13713, Saudi Arabia
8 Department of Pharmacognosy, Faculty of Pharmacy, Al-Azhar University, Cairo, 11371, Egypt
* Correspondence: laridafarida@umc.edu.dz (F.L.); mghoneim@mcst.edu.sa (M.M.G.), Tel.: +966-537415011 (F.L. & M.M.G.).

Table of Content

	Page
**Figure S1. ^{1}H NMR spectrum of compound 1.**	**3**
**Figure S2.^{13}C NMR spectrum of compound 1.**	**4**
**Figure S3. HRESIMS (-) for compound 1.**	**5**
**Figure S4. ^{1}H NMR spectrum of compound 2.**	**6**
**Figure S5. ^{13}C NMR spectrum of compound 2**	**7**
**Figure S6. HRESIMS (-) for compound 2**	**8**
**Figure S7. ^{1}H NMR spectrum of compound 3.**	**9**
**Figure S8. ^{13}C NMR spectrum of compound 3**	**10**
**Figure S9. HRESIMS (-) for compound 3**	**11**
**Figure S10. ^{1}H NMR spectrum of compound 4.**	**12**
**Figure S11. ^{13}C NMR spectrum of compound 4**	**13**
**Figure S12. HRESIMS (-) for compound 4**	**14**
**Figure S13. ^{1}H NMR spectrum of compound 5.**	**15**
**Figure S14. ^{13}C NMR spectrum of compound 5**	**16**
**Figure S15. HRESIMS (-) for compound 5**	**17**
**Figure S16. ^{1}H NMR spectrum of compound 6**	**18**
**Figure S17. ^{13}C NMR spectrum of compound 6**	**19**
**Figure S18. HRESIMS (-) for compound 6**	**20**
**Figure S19. ^{1}H NMR spectrum of compound 7**	**21**
**Figure S20. ^{13}C NMR spectrum of compound 7**	**22**
**Figure S21. HRESIMS (-) for compound 7**	**23**

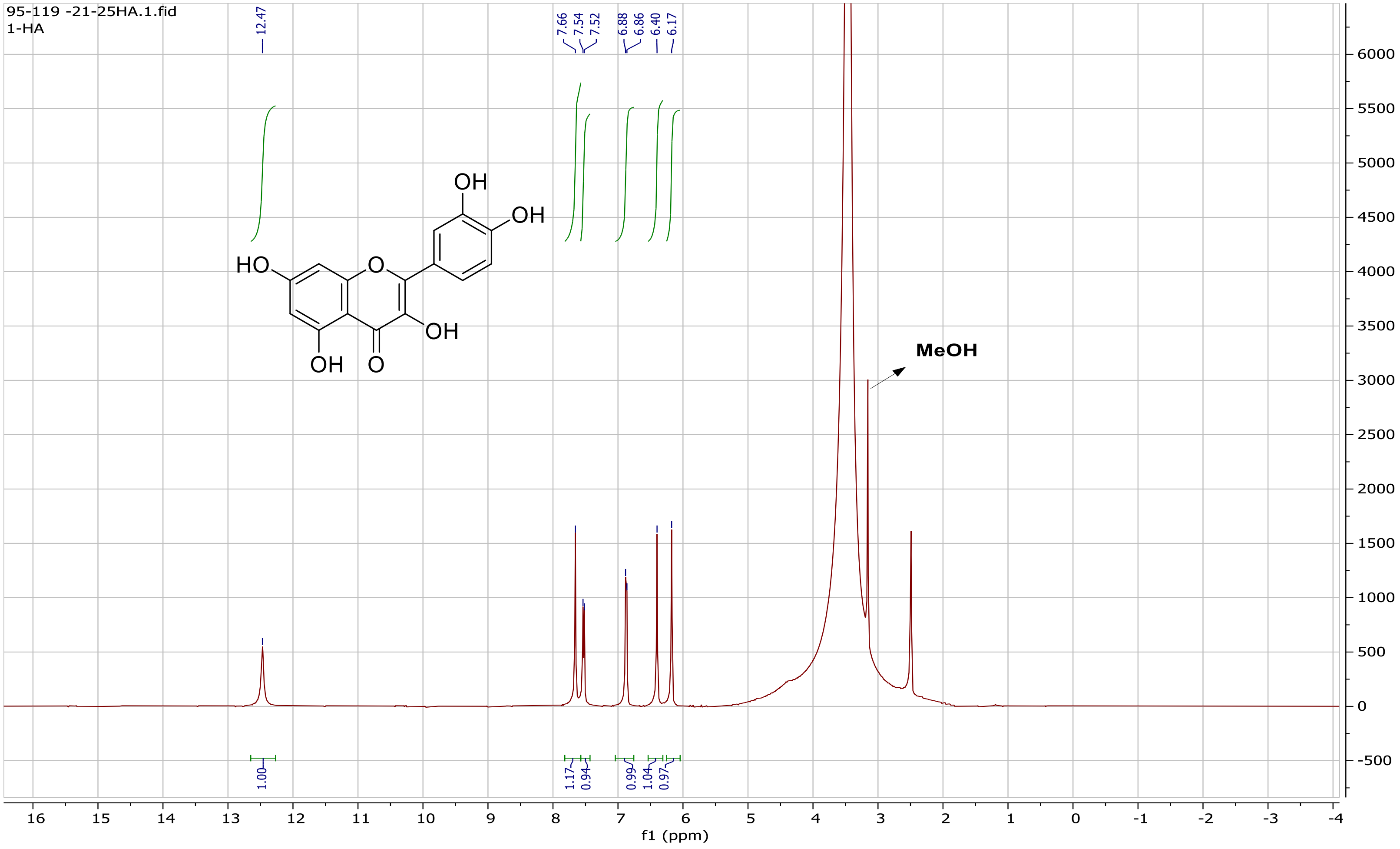

Figure S1. **^{1}H NMR spectrum of compound 1 (DMSO-d_6, 400 MHz)**

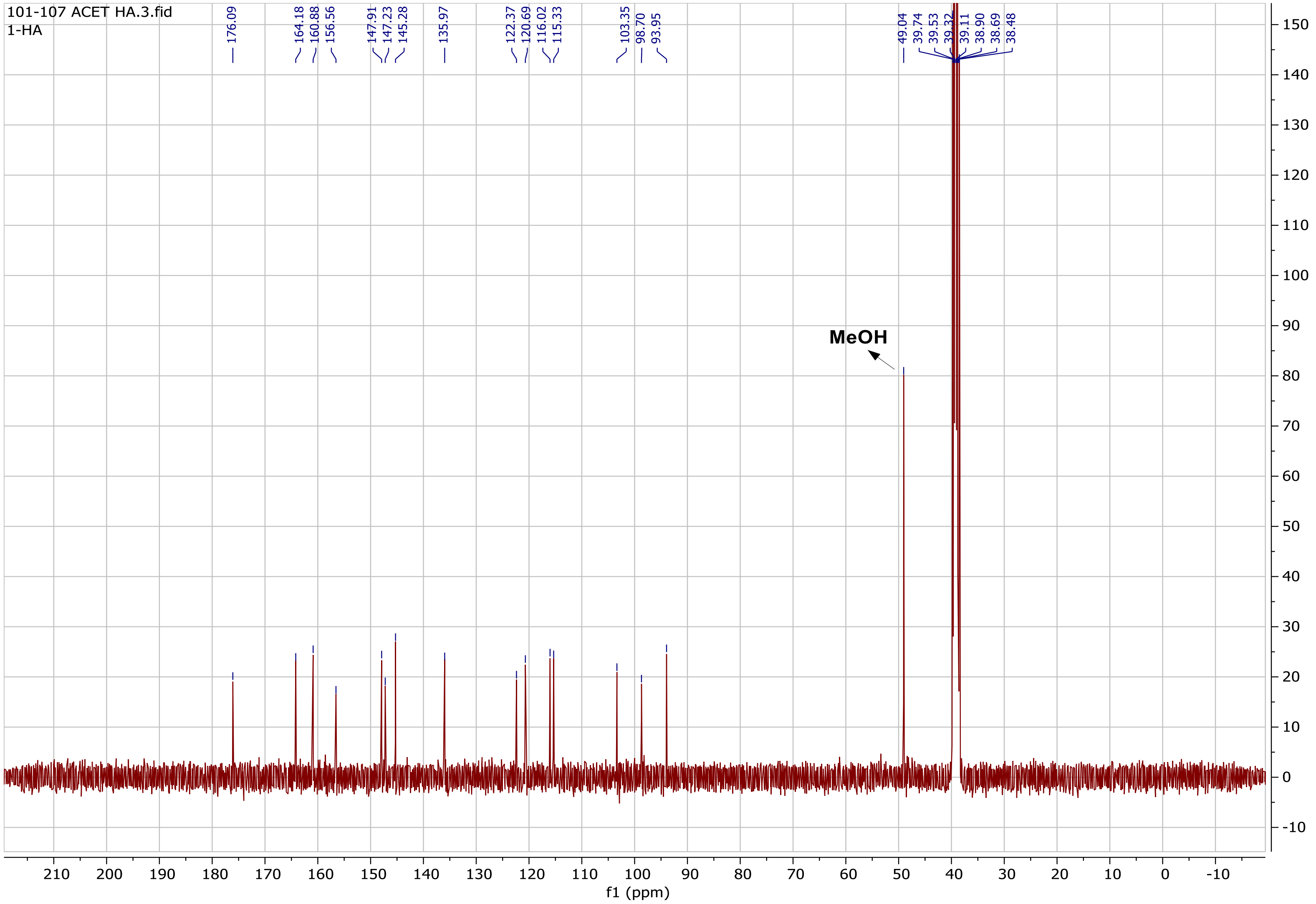

Figure S2. ^{13}**C NMR spectrum of compound 1 (DMSO-d$_6$, 100 MHz)**

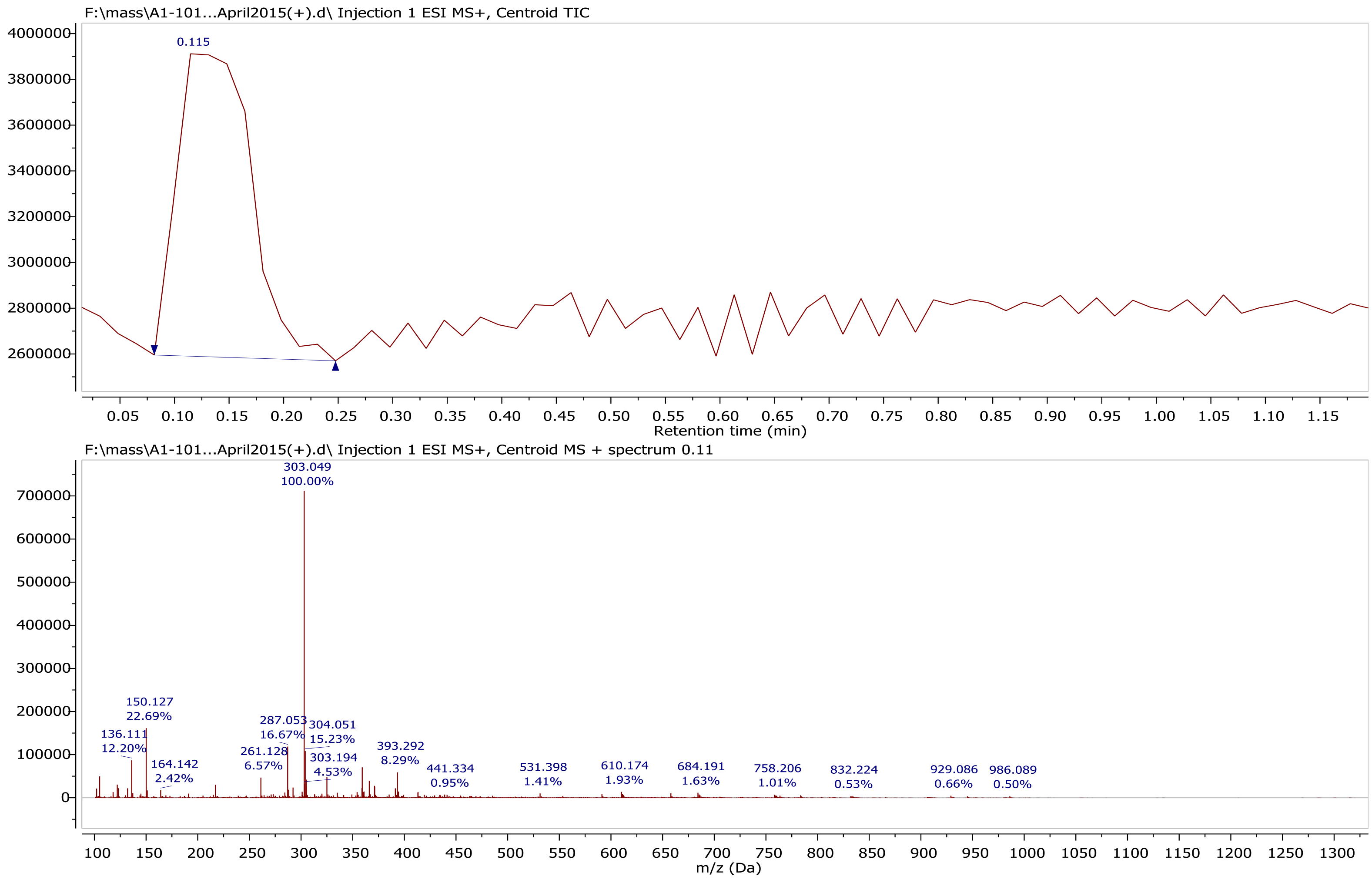

Figure S3. Positive HRESIMS of compound 1

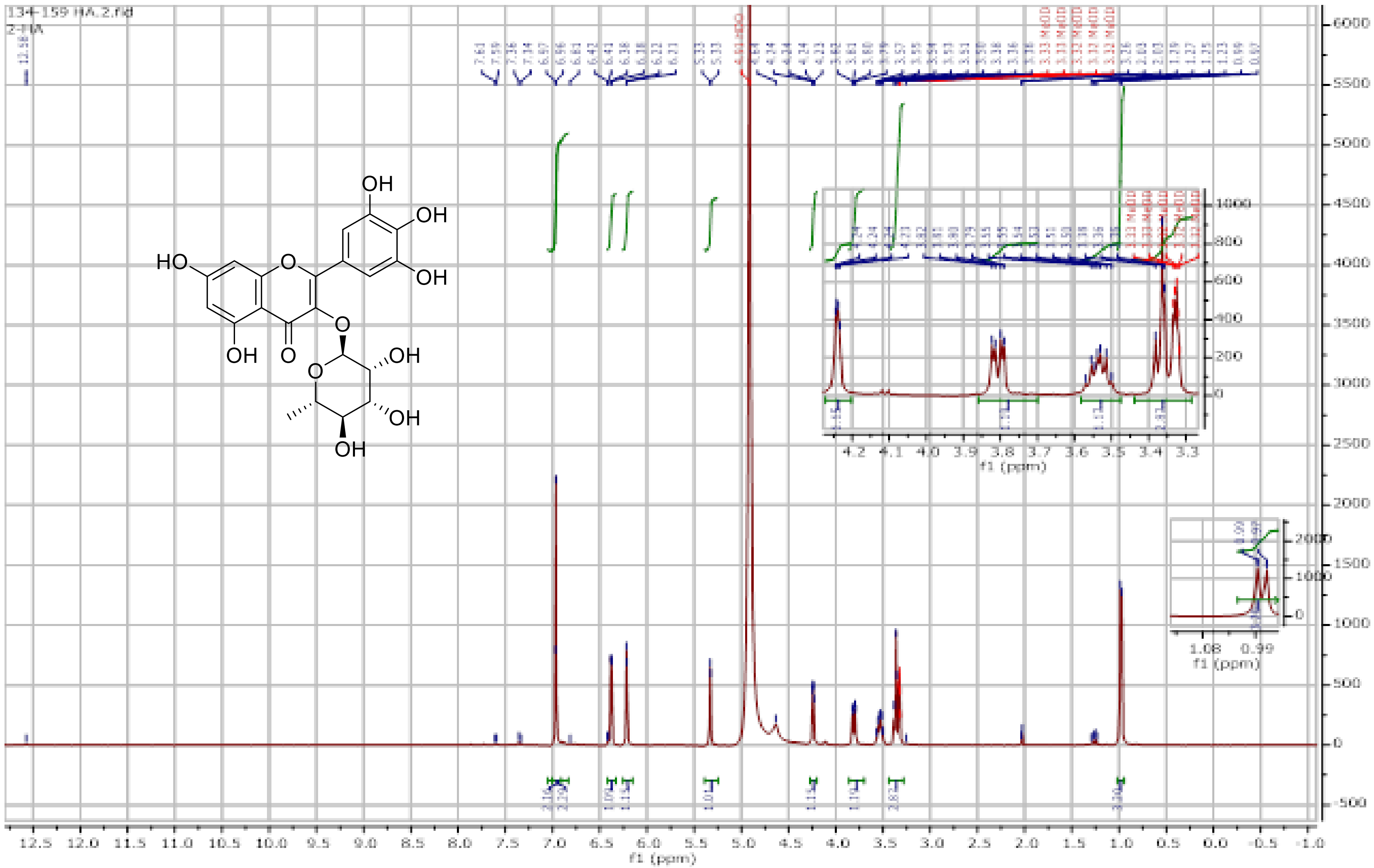

Figure S4. ^{1}H NMR spectrum of compound 2 (Methanol-d_4, 400 MHz)

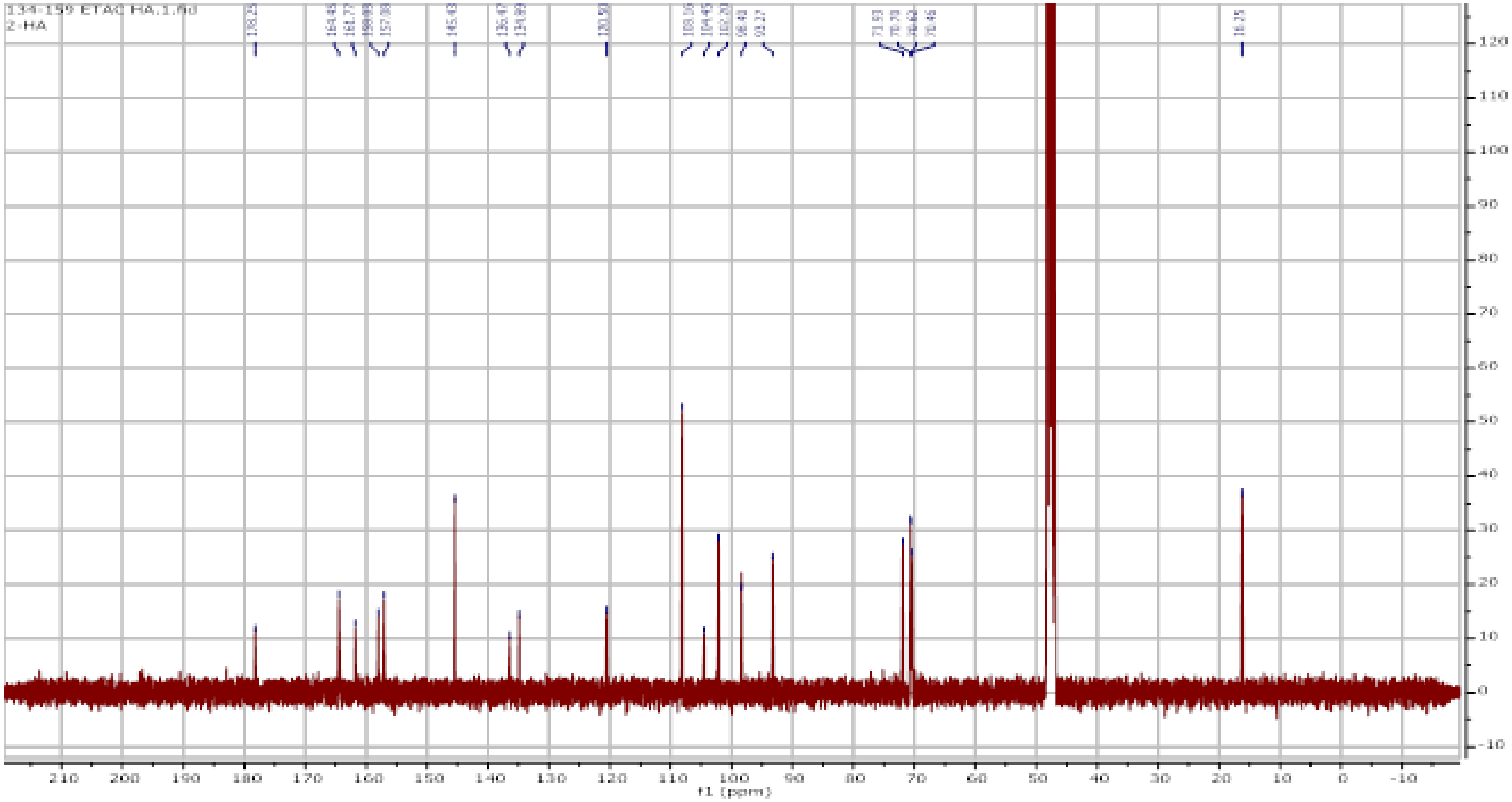

Figure S5. **^{13}C NMR spectrum of compound 2 (Methanol-*d_4*, 100 MHz)**

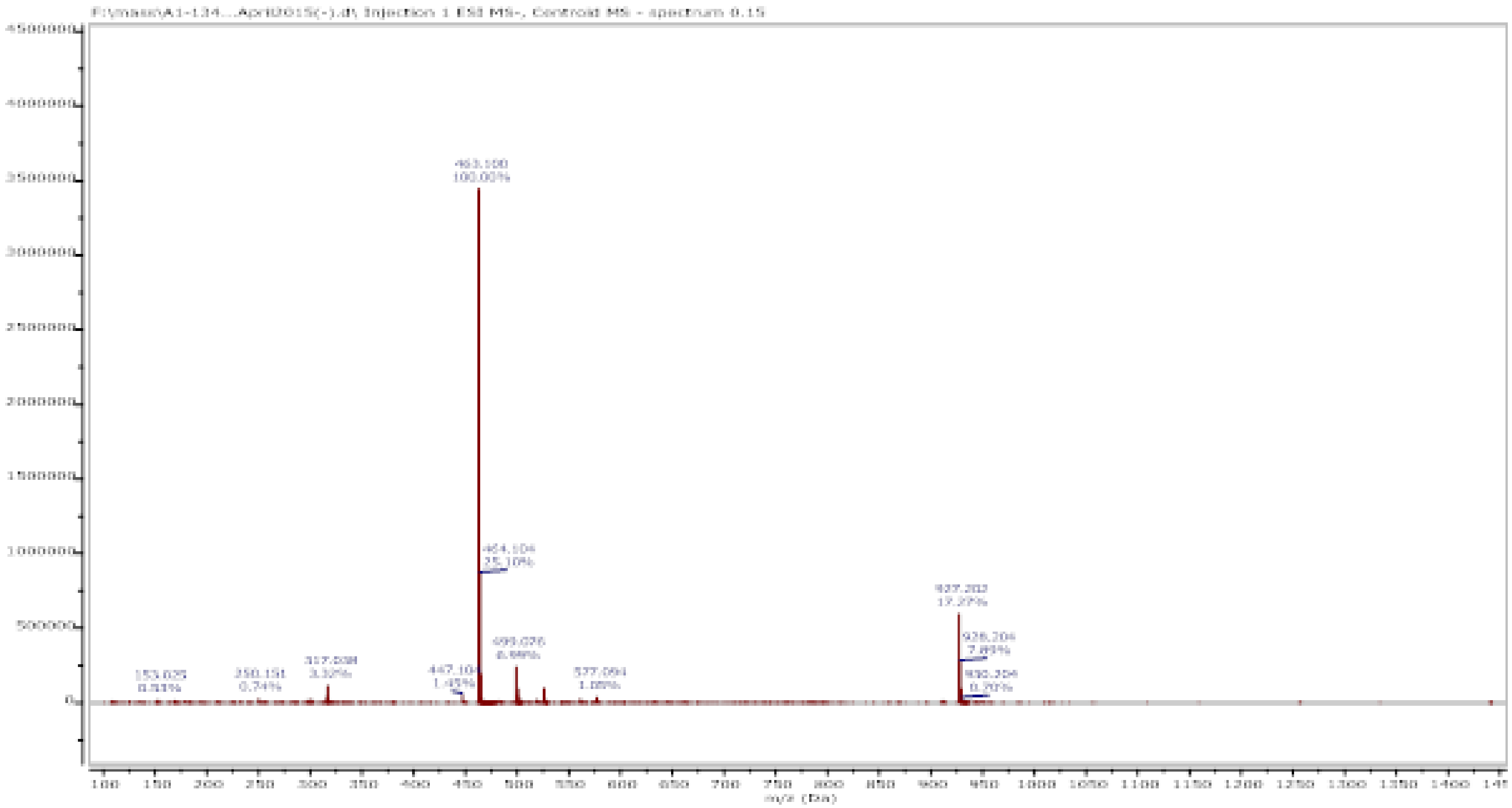

Figure S6. Negative HRESIMS of compound 2

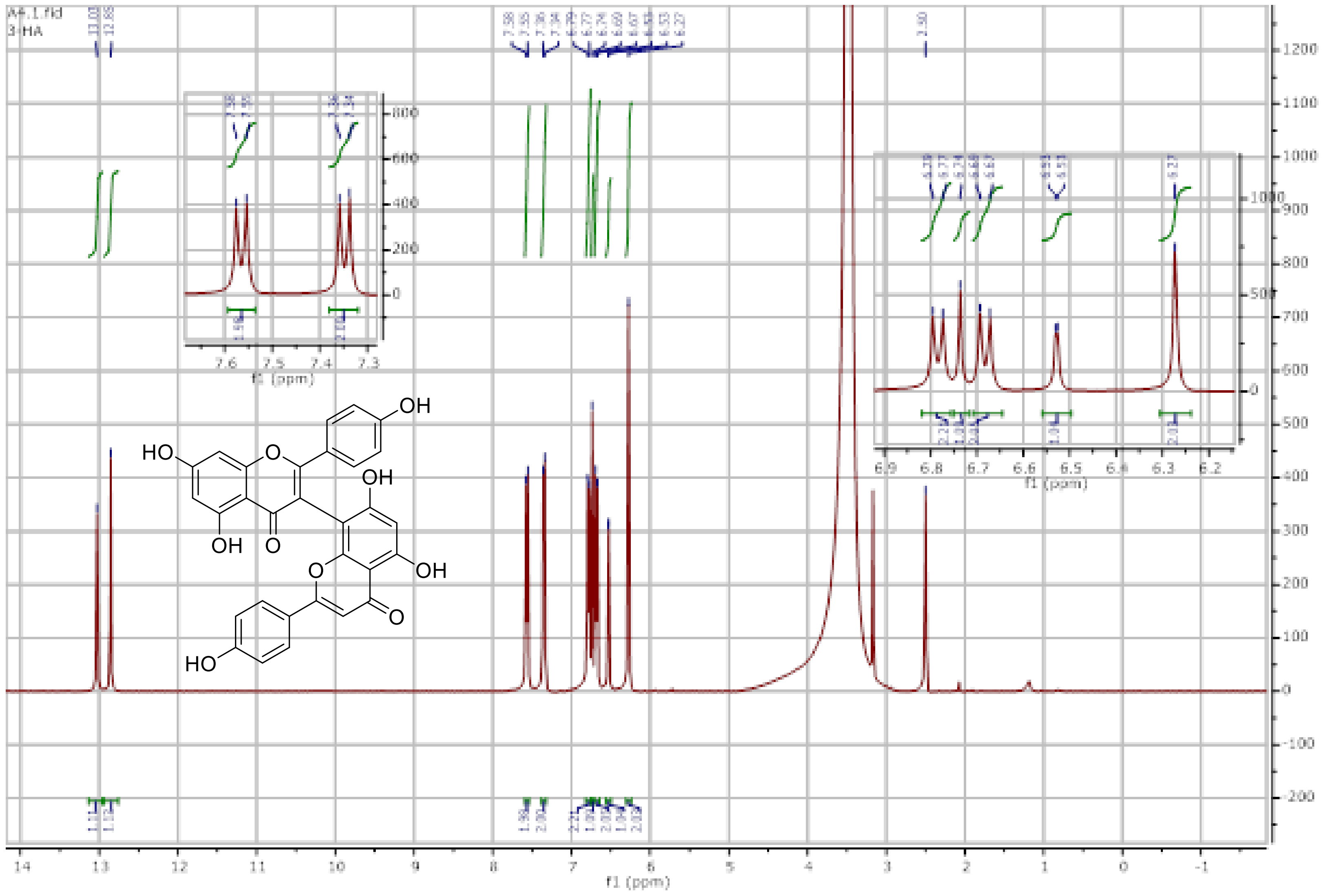

Figure S7. ^{1}H NMR spectrum of compound 3 (DMSO-d_6, 400 MHz)

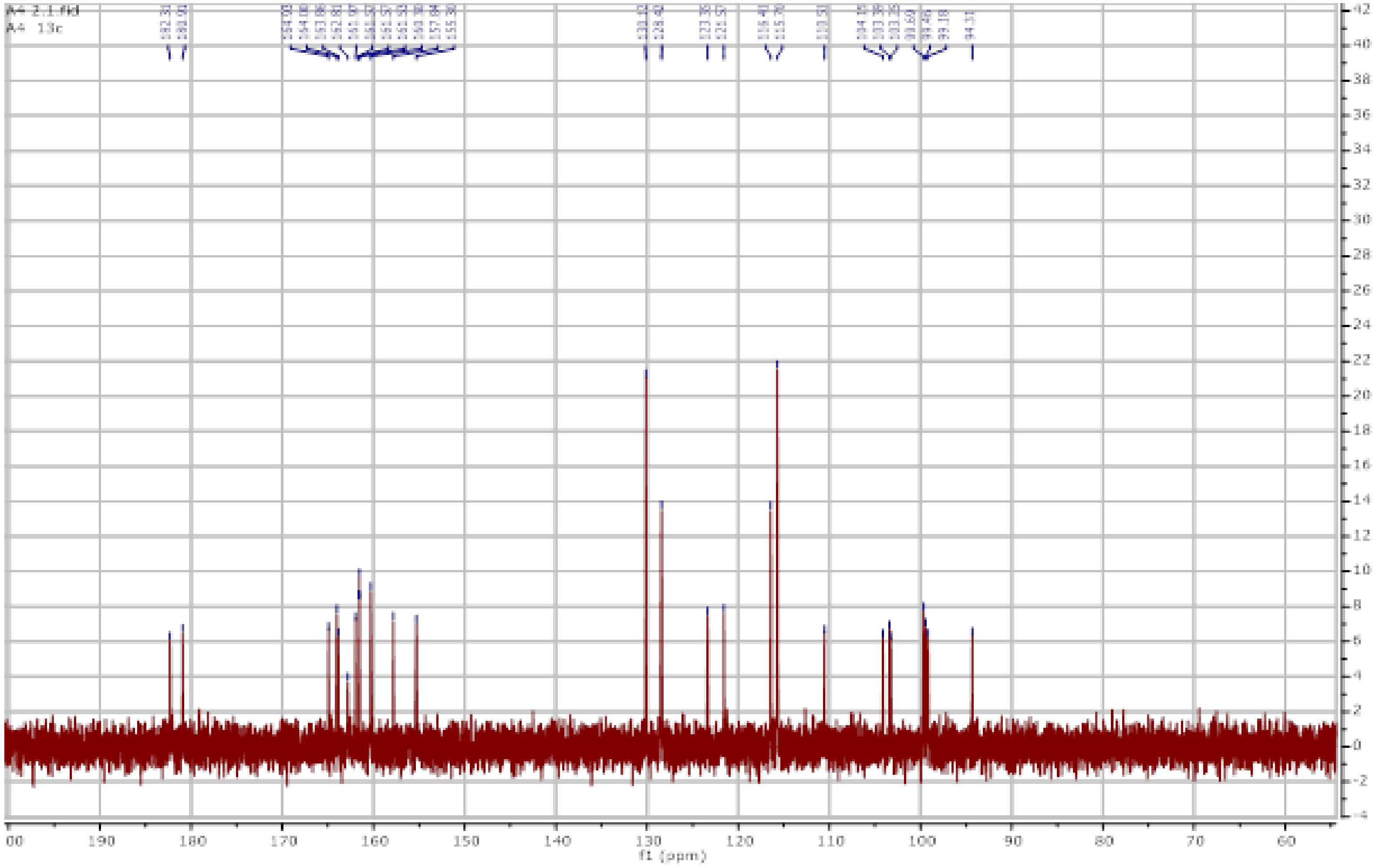

Figure S8. ^{13}C NMR spectrum of compound 3 (DMSO-d_6, 100 MHz)

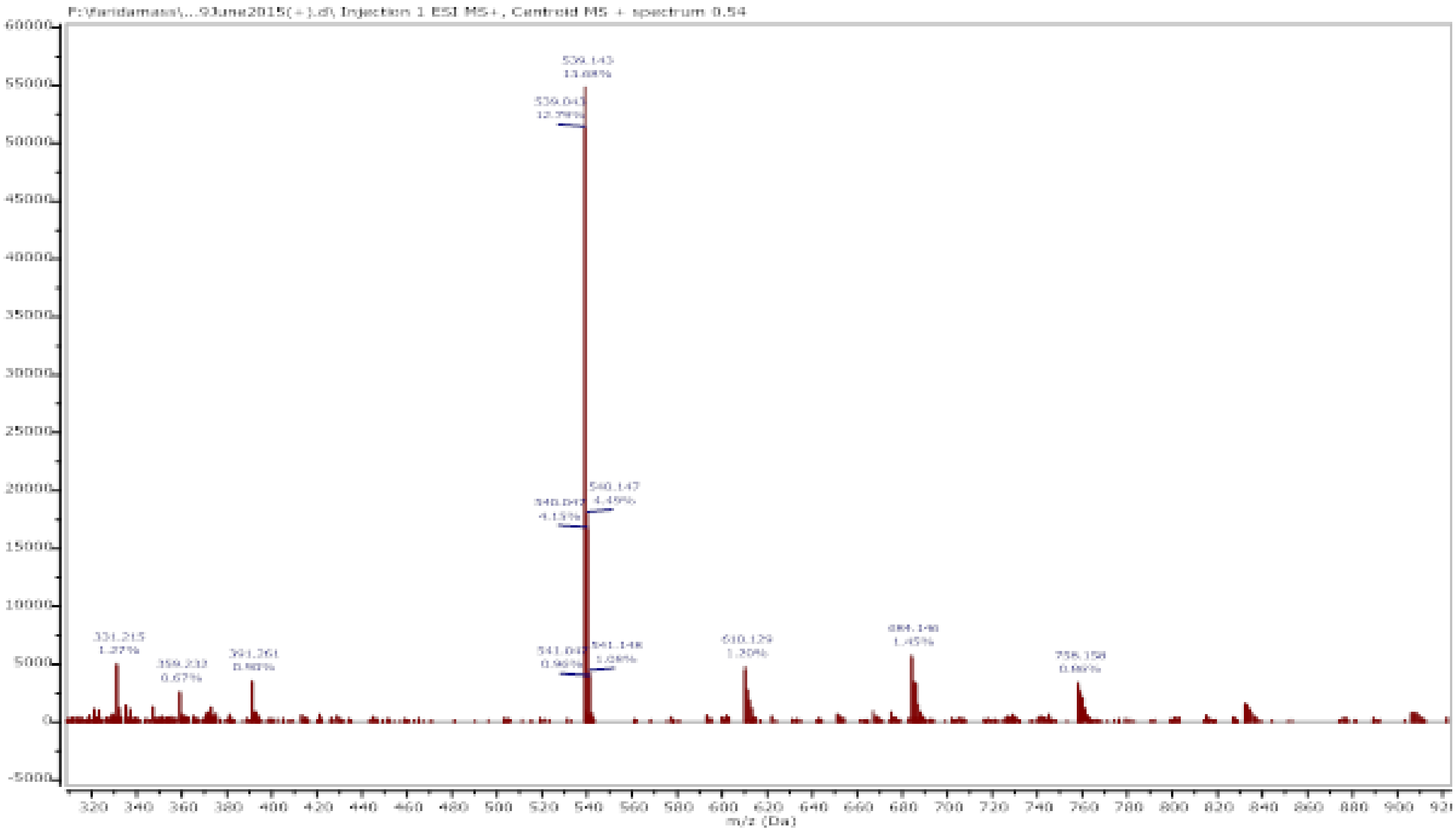

Figure S9. Positive HRESIMS of compound 3

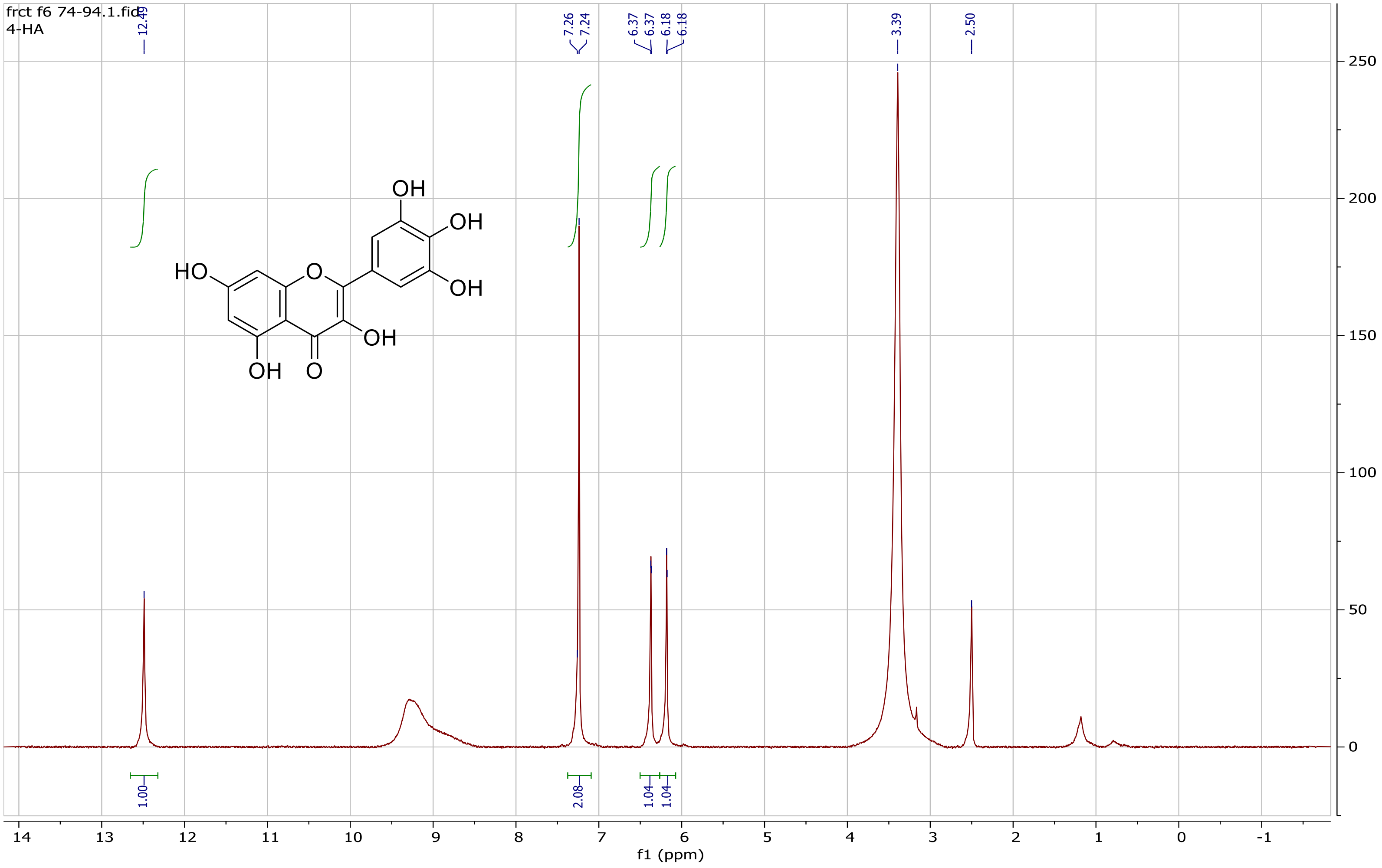

Figure S10. ^{1}H NMR spectrum of compound 4 (DMSO-d_6, 400 MHz)

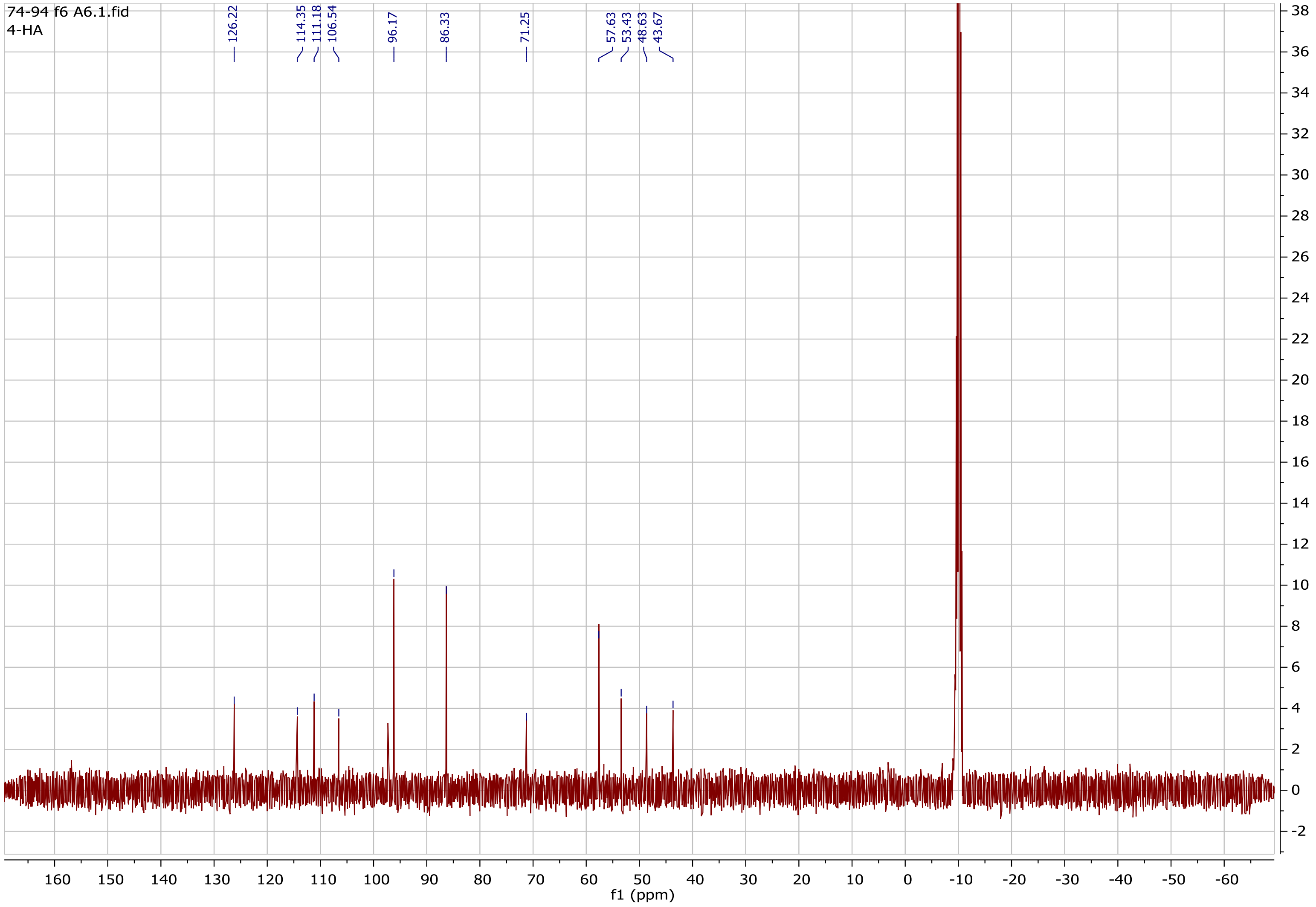

Figure S11. ^{13}**C NMR spectrum of compound 4 (DMSO-*****d*****$_6$, 400 MHz)**

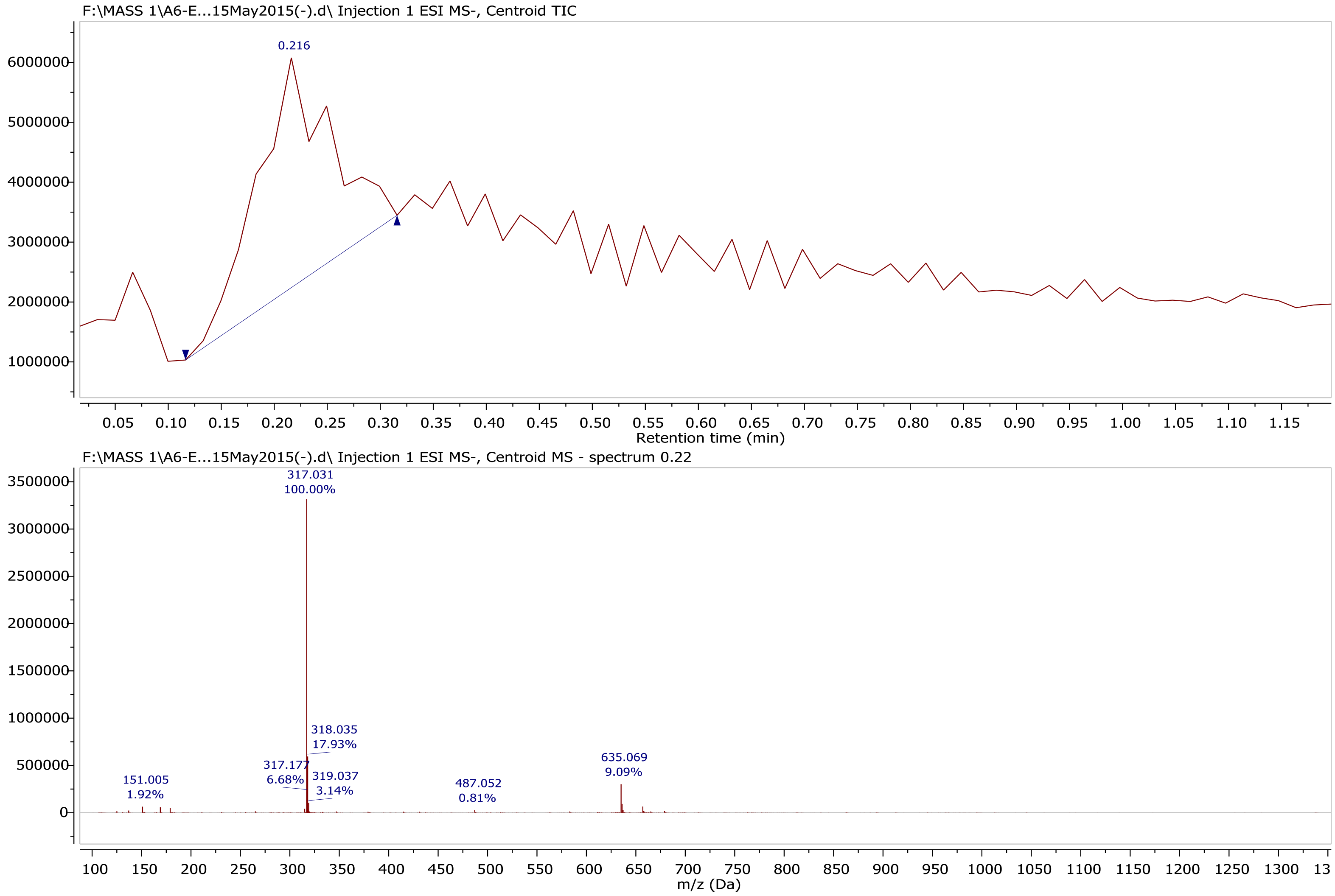

Figure S12. HRESIMS (-) for compound 4

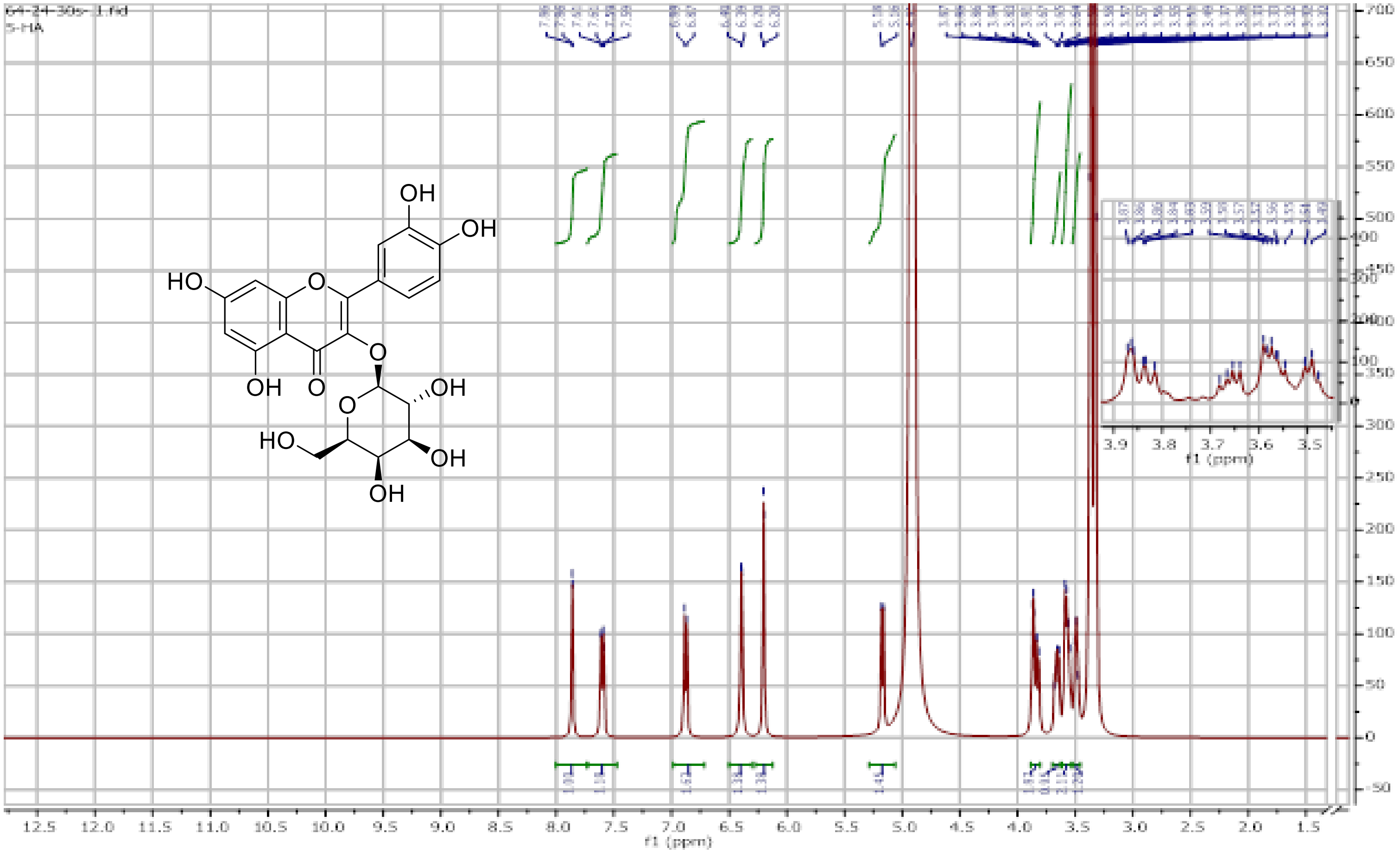

Figure S13. **^{1}H NMR spectrum of compound 5 (DMSO-d_6, 400 MHz)**

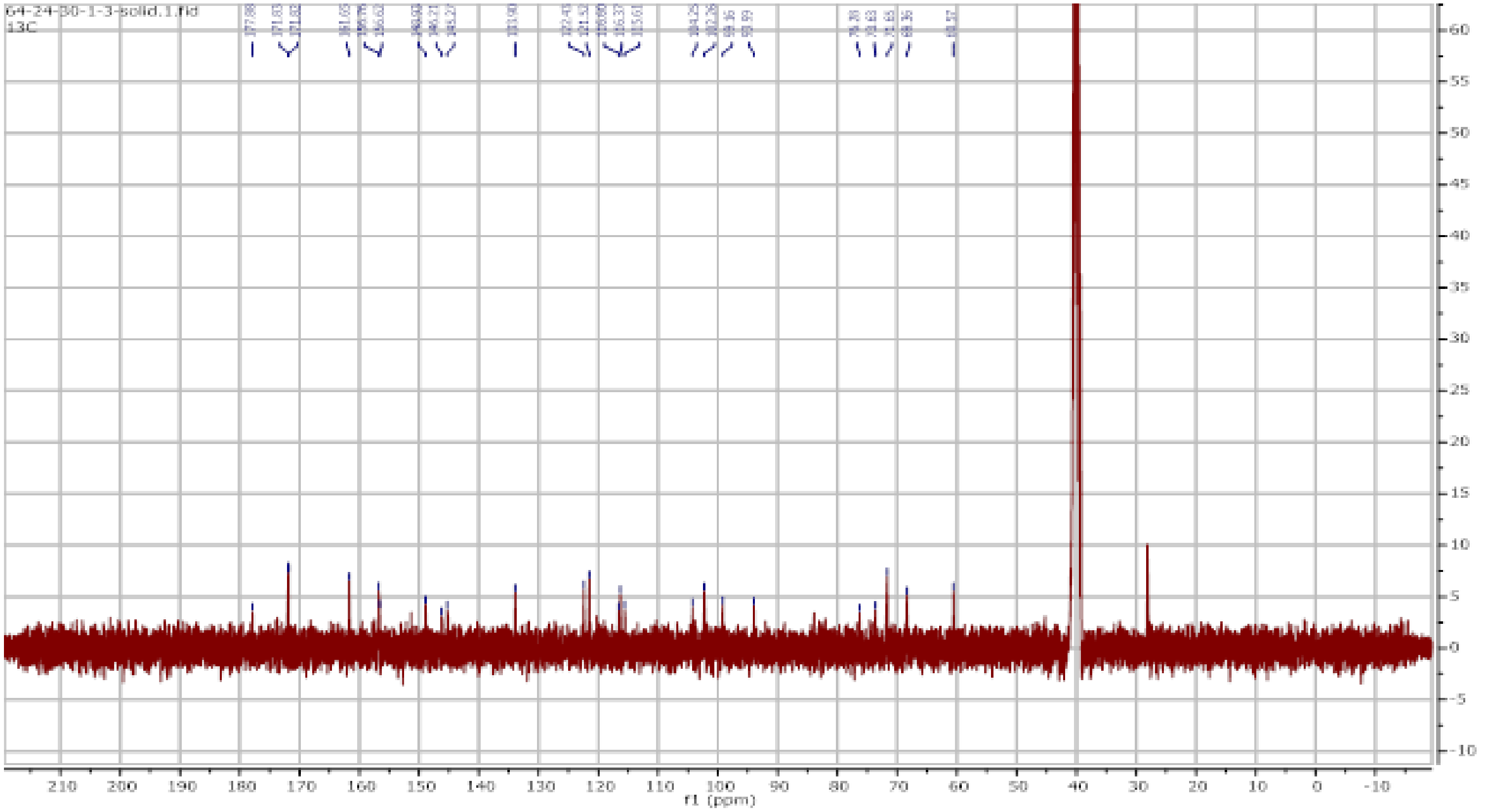

Figure S14. ^{13}C NMR spectrum of compound 5 (DMSO-d_6, 100 MHz)

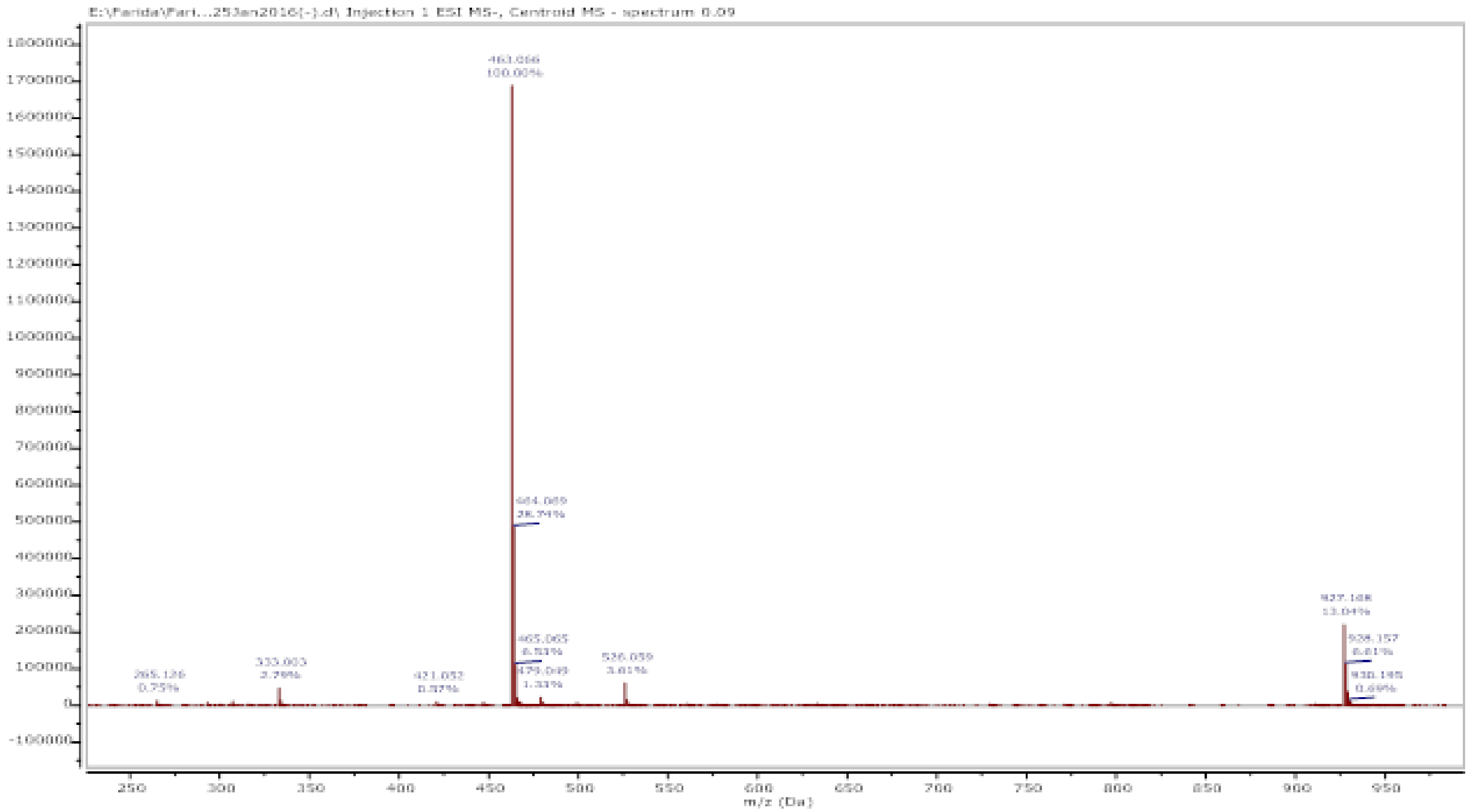

Figure S15. HRESIMS (-) for compound 5

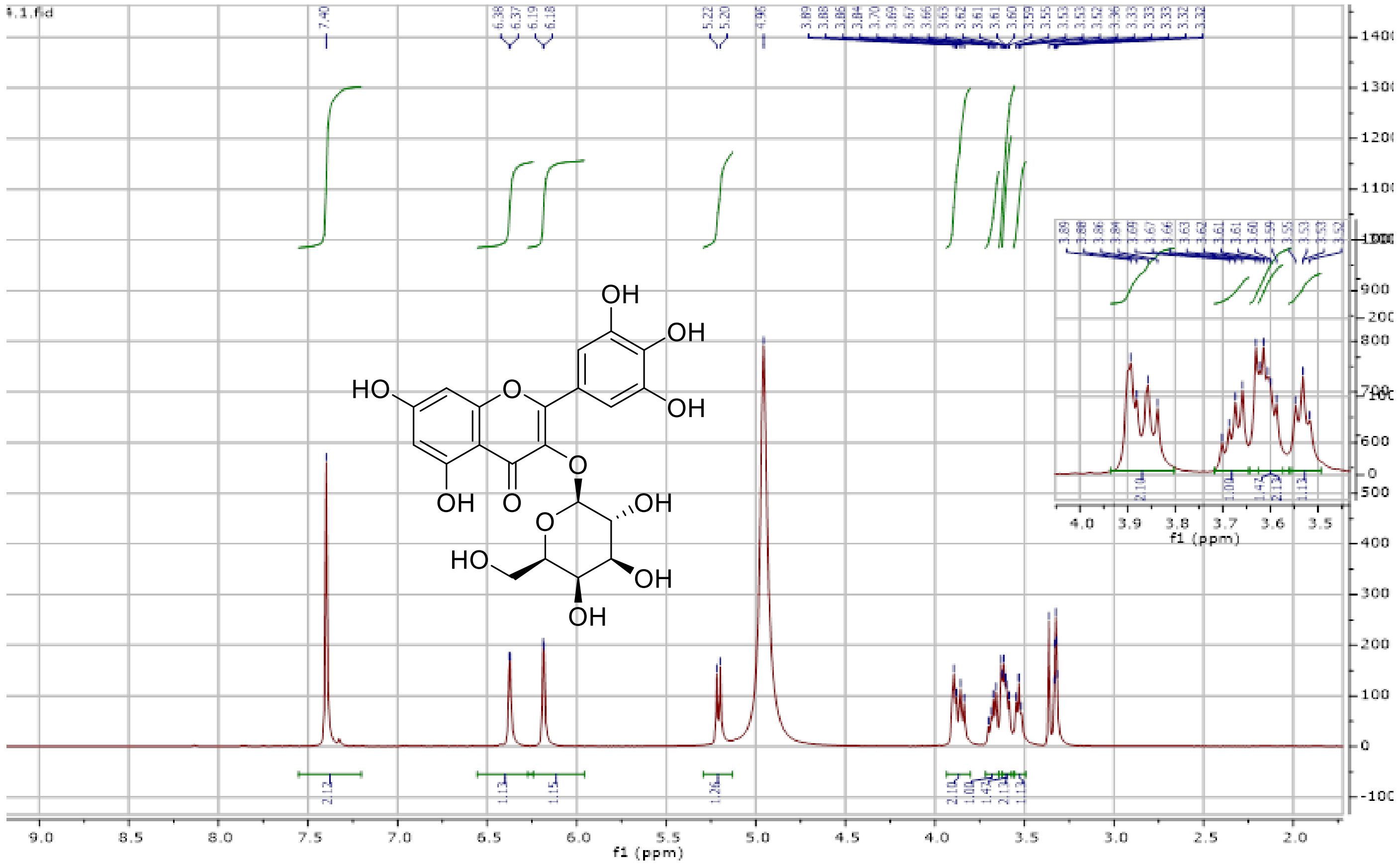

Figure S16. ^{1}H NMR spectrum of compound 6 (Methanol-d_4, 400 MHz)

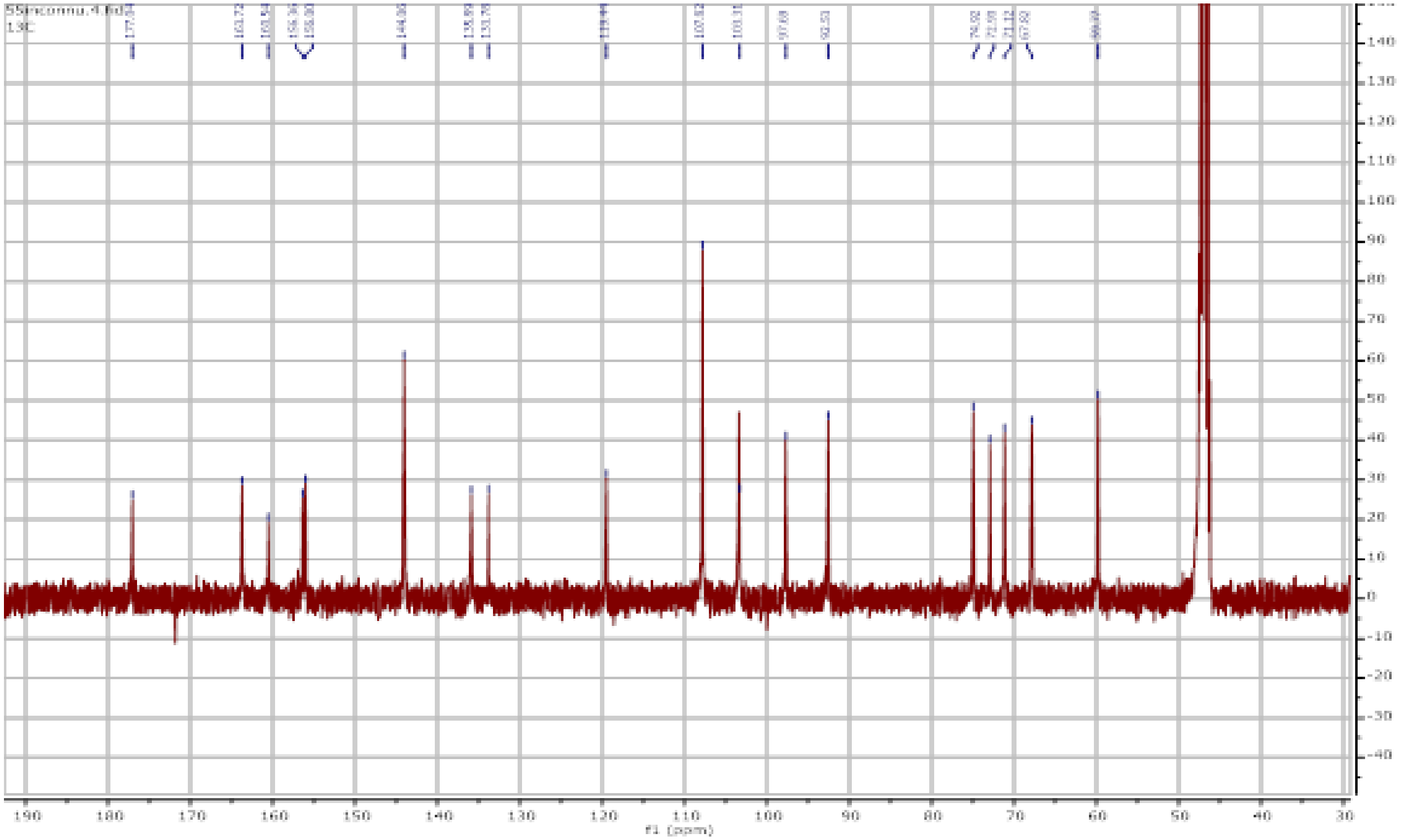

Figure S17. ^{13}C NMR spectrum of compound 6 (Methanol-d_4, 100 MHz)

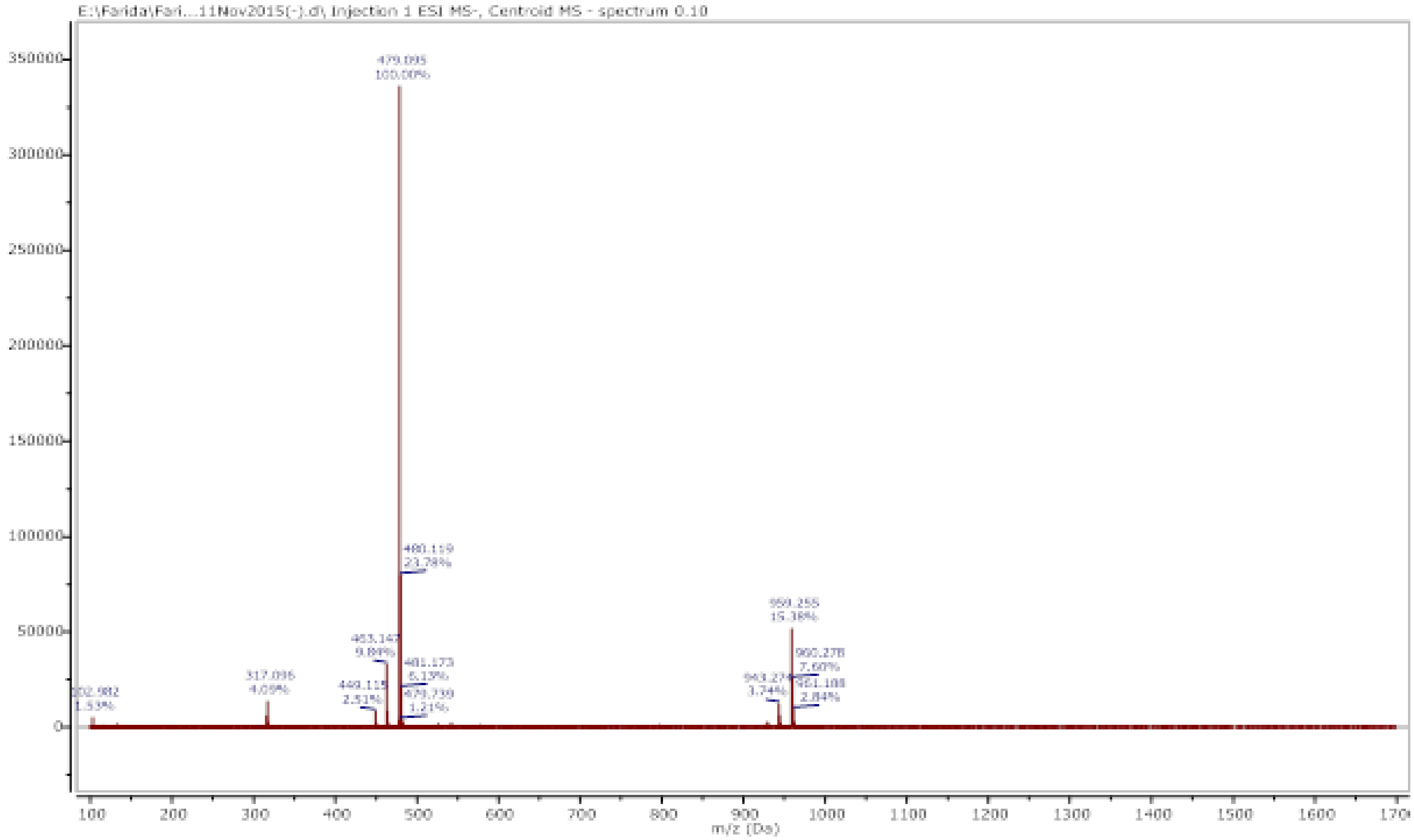

Figure S18. HRESIMS (-) for compound 6

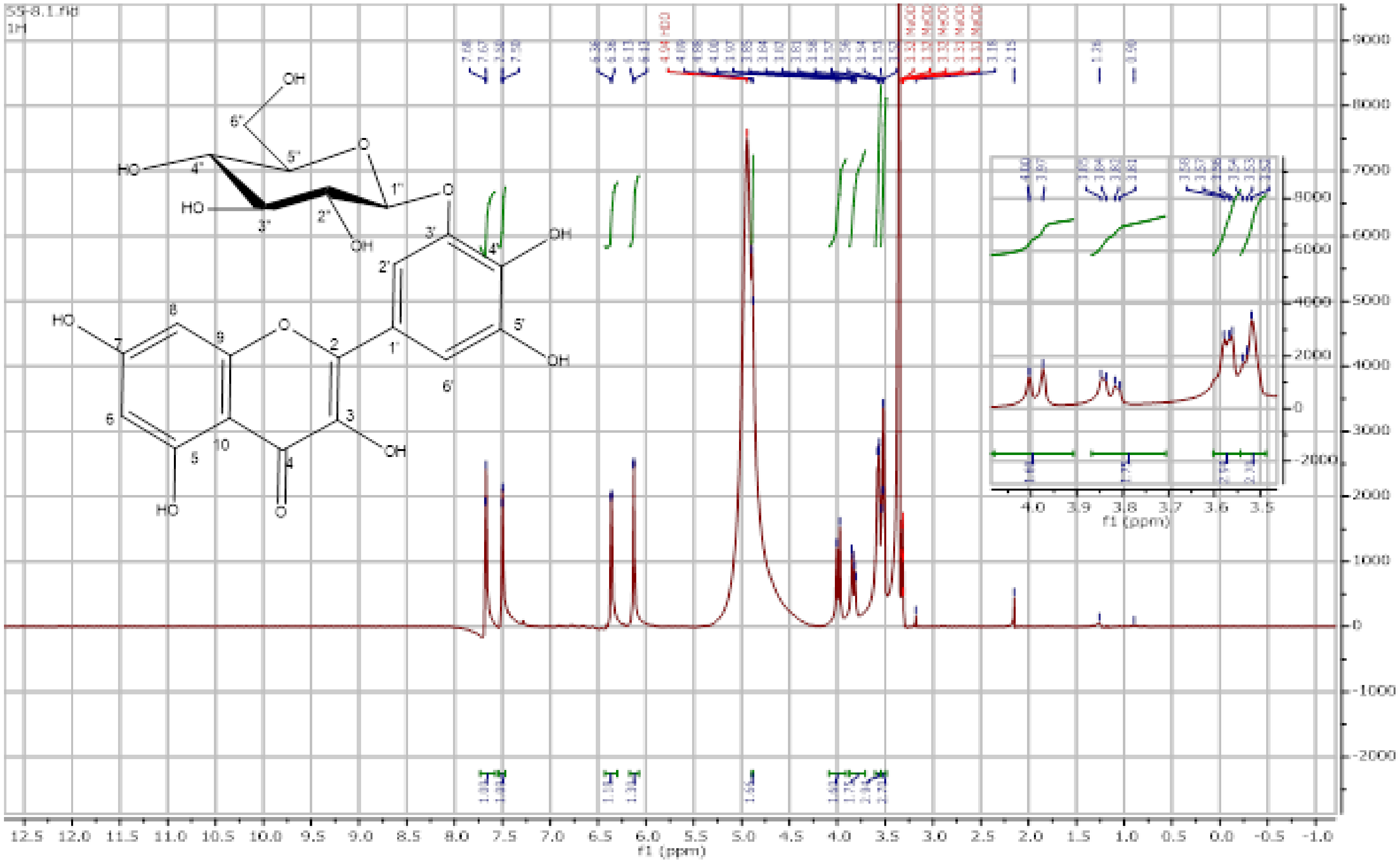

Figure S19. ^{1}H NMR spectrum of compound 7 (Methanol-d_4, 400 MHz)

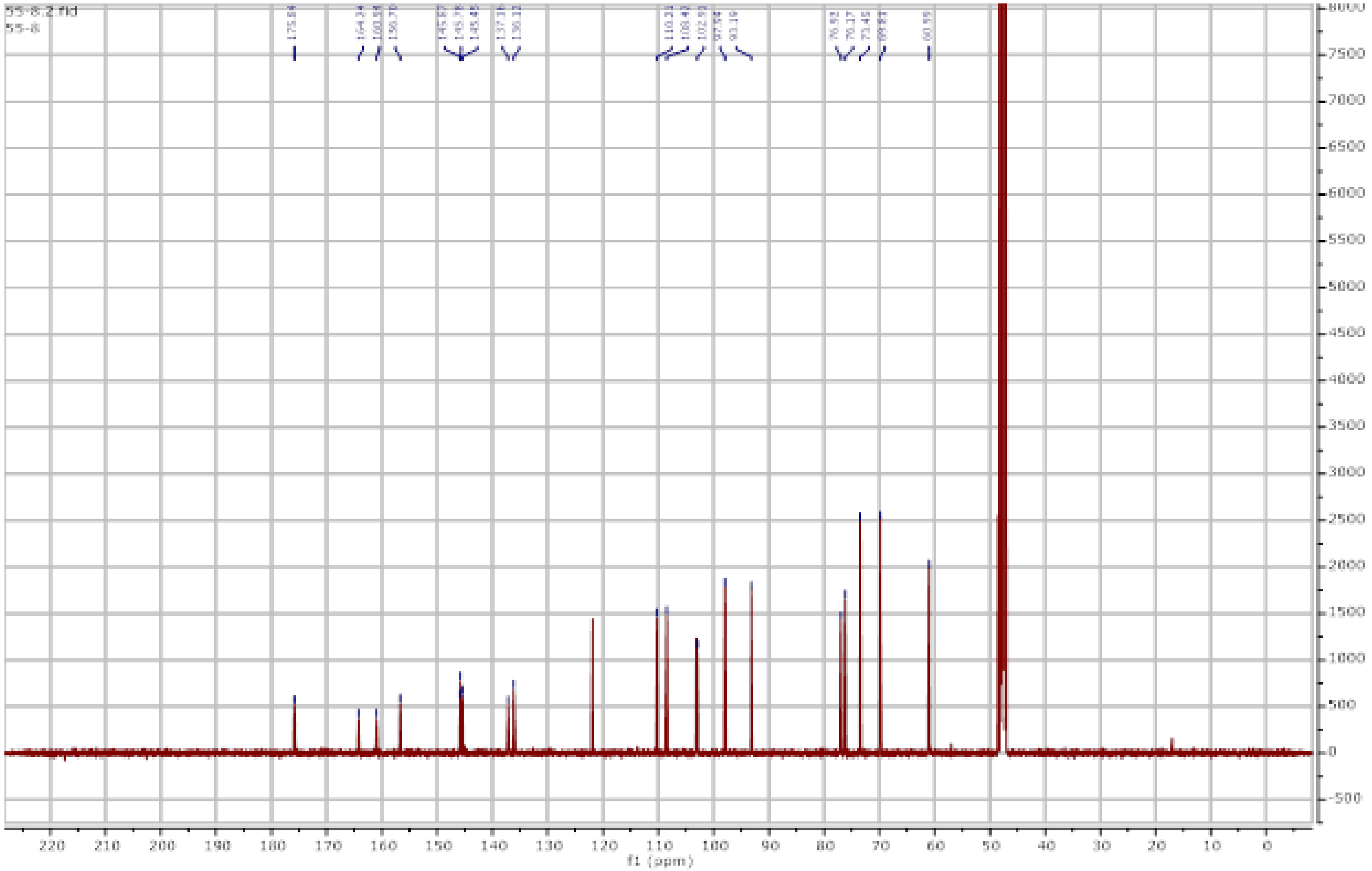

Figure S20. [13]**C NMR spectrum of compound 7 (Methanol-d**$_4$**, 100 MHz)**

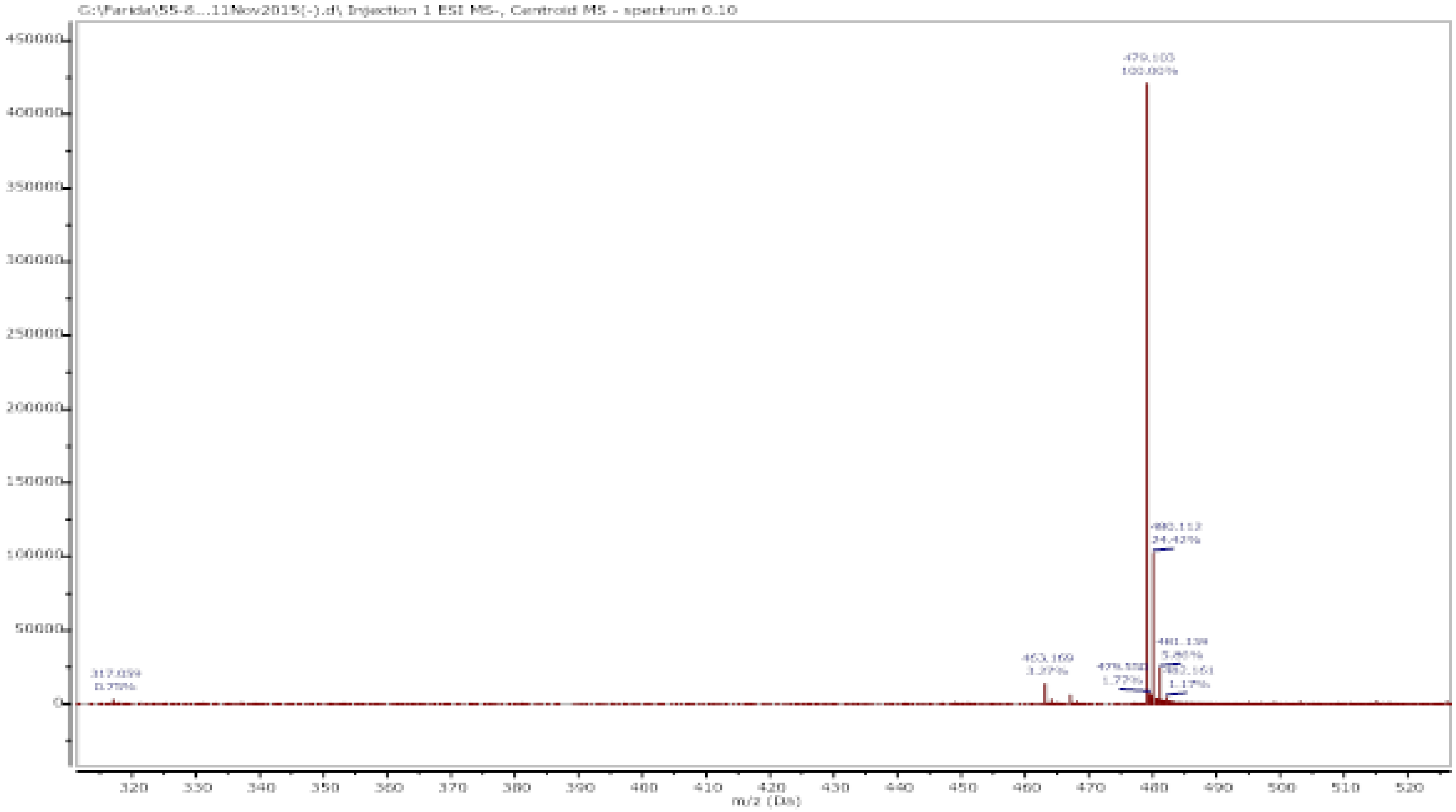

Figure S21. HRESIMS (-) for compound 7
